# Occurrence, variability and evolutionary relations of efflux pump determinants across five pathogenic *Staphylococcus* species

**DOI:** 10.3389/fphar.2026.1870078

**Published:** 2026-09-18

**Authors:** Carolina Ferreira, Diana Martinho, Ricardo Parreira, José Melo-Cristino, Miguel Viveiros, Sofia Santos Costa, Isabel Couto

**Affiliations:** 1 Global Health and Tropical Medicine, GHTM, LA-REAL, Instituto de Higiene e Medicina Tropical, IHMT, Universidade NOVA de Lisboa, Lisbon, Portugal; 2 Laboratório de Microbiologia, Serviço de Patologia Clínica, Centro Hospitalar Universitário Lisboa Norte, Lisboa, Portugal; 3 Instituto de Microbiologia, Faculdade de Medicina, Universidade de Lisboa, Lisbon, Portugal

**Keywords:** allele, efflux, genetic diversity, multidrug transporters, phylogenetic analysis

## Abstract

Multidrug efflux pumps (MDR EPs) play a major role in the emergence of antimicrobial resistance in *Staphylococcus aureus*. Among these, the native NorA is the most well-studied MDR EP in this species. However, efflux-mediated resistance is only scarcely characterized in other staphylococci. The aims of this study were to assess the genetic variability and phylogenetic relations of main efflux pump genes in *S. aureus* and four other pathogenic staphylococci, and to identify new putative MDR transporters in these species. Nucleotide sequences of *norA*, *norB*, *norC*, *norB/C*, *mepA* and related sequences of *S. aureus*, *Staphylococcus epidermidis*, *Staphylococcus haemolyticus*, *Staphylococcus lugdunensis* and *Staphylococcus hominis* were compiled and assessed for gene variability. The *norA* variability was further evaluated in a collection of clinical strains of *S. epidermidis*, *S. haemolyticus* and *S. hominis*. Phylogenetic analyses were performed to characterize the evolution of *norA* across the five species and to explore potential relations between *norB*, *norC*, *norB/C* and related sequences. Additional putative MDR transporter genes were searched in public databases and then BLAST searched against complete genomes of each species. Only *S. aureus* presented several *norA* and *norB* alleles. The relations between the *norA* genes of the five species follow the evolutionary relations of these staphylococci. Phylogenetic analysis also revealed the existence of a *norB/C* gene in *S. aureus*, which is homologous to the *norB/C* genes of *S. epidermidis*, *S. haemolyticus*, *S. lugdunensis* and *S. hominis*. It also indicated that *S. aureus norB* and *norC* as well as the newly assigned *S. haemolyticus norB/C-like* are related genes, absent in the other species. *In silico* analysis identified several new candidate MDR transporters in the five species. This study provides a novel, comprehensive comparative analysis of major efflux pump determinants across five clinically relevant *Staphylococcus* species. This analysis expanded our knowledge of the *norA* evolutionary pathways and provided a framework for a more consistent annotation of *norB*, *norC* and related determinants among these staphylococci. The identification of putatively new MDR transporter determinants in staphylococcal species of clinical relevance other than *S. aureus* provides a framework for future functional studies.

## 1 Introduction

Efflux pumps are membrane proteins that are present in all organisms, involved in the export of different molecules from within the cell to the external environment. Although efflux activity is often associated with antimicrobial resistance, these systems primarily serve other physiological roles, essential for cell survival and drug efflux is likely a secondary function. Efflux pumps may remove metabolic waste products or other endogenously produced metabolites that are noxious to the cell in excess (Henderson et al., 2021). They are also important for bacterial infection and persistence in the host, exporting virulence determinants, including adhesins, toxins and other proteins that are important for colonization of host cells, response to oxidative stress, efflux of quorum-sensing molecules and maintenance of iron homeostasis or biofilm formation (Resch et al., 2005; Deng et al., 2012; Rumbo-Feal et al., 2013; Huang et al., 2022; Truong-Bolduc et al., 2024; Truong-Bolduc et al., 2025). They can also confer resistance to natural substances produced by the host, including bile, hormones and host-defense molecules that act as antimicrobials (Piddock, 2006; Henderson et al., 2021).

Efflux pumps can be specific, exporting only one antimicrobial or antimicrobials from the same class. Their genes are mostly located on plasmids or other mobile genetic elements, although some can be chromosomally encoded. Other efflux pumps export several structurally different molecules, including antibiotics of different classes or biocides. These are associated with multidrug resistance phenotypes and referred to as multidrug efflux pumps (MDR EPs). There are five main classes of bacterial MDR EPs, classified according to structural differences, sources of energy and substrate specificity: the ATP-binding cassette (ABC) superfamily, the major facilitator superfamily (MFS), the resistance nodulation division (RND) superfamily, the multidrug and toxic-compound extrusion (MATE) family, which is part of the multidrug/oligosaccharidyl-lipid/polysaccharide (MOP) flippase superfamily, and the small multidrug resistance (SMR) family, which is part of the drug/metabolite transporter superfamily (Piddock, 2006). Two additional families of transporters were described more recently: the proteobacterial antimicrobial compound efflux family (Hassan et al., 2018) and the p-aminobenzoyl-glutamate transporter family (Delmar and Yu, 2016).

*Staphylococcus* *aureus* is a major human pathogen responsible for severe infections in both healthcare and community settings. The treatment of these infections, particularly those caused by methicillin-resistant *S. aureus* (MRSA) strains, is increasingly challenging (De Oliveira et al., 2020), which has led to its classification as a high-priority pathogen by the World Health Organization (World Health Organization, 2024). In parallel, antimicrobial resistance (AMR) is also rising among coagulase-negative staphylococci (CoNS), including *Staphylococcus epidermidis*, *Staphylococcus haemolyticus*, *Staphylococcus hominis* and *Staphylococcus lugdunensis*, which are gaining clinical relevance as opportunistic pathogens (Becker et al., 2020).

Among CoNS, *S. epidermidis* is the leading cause of bloodstream infections, associated with the use of medical devices, largely due to its ability to form biofilms and persist in hospital environments (Sabaté Brescó et al., 2017). Its long-term adaptation has driven the accumulation of resistance and virulence-associated genes, contributing to the emergence of multidrug-resistant strains, including methicillin-resistant *S. epidermidis* (Rolo et al., 2026). Similarly, *S. haemolyticus* and *S. hominis* are important opportunistic pathogens, also associated with the use of implanted medical devices, particularly in immunocompromised patients. *S. haemolyticus* is the second CoNS most frequently isolated from blood cultures and ocular infections (Panda and Singh, 2018; Pain et al., 2019; Becker et al., 2020) and exhibits high rates of antimicrobial resistance that limit treatment options (Becker et al., 2020; Meenatchi et al., 2025). *S. hominis* is the third CoNS most often isolated from bloodstream infections and is increasingly associated with methicillin and multidrug resistance (Szczuka et al., 2018; Szemraj et al., 2020). Although *S. lugdunensis* remains generally more susceptible to antibiotics, methicillin resistance in this species has been rising in recent years (Becker et al., 2020; Heilbronner and Foster, 2020). Skin and soft tissue infections are the most common infections caused by *S. lugdunensis*, but it can cause severe invasive infections, including native valve endocarditis and bacteremia (Becker et al., 2020; Michels et al., 2021).

Efflux pumps are key contributors to antimicrobial resistance in staphylococci. In *S. aureus*, more than 20 efflux systems have been described, including major multidrug efflux pumps such as NorA, NorB, NorC, MepA, MdeA, LmrS and SdrM, as well as plasmid-encoded systems like QacA/B and QacC (Kadlec et al., 2012; Costa et al., 2013; Dashtbani-Roozbehani and Brown, 2021). While extensively studied in *S. aureus*, efflux-mediated resistance remains poorly characterized in other clinically relevant staphylococci, particularly regarding the genetic diversity and evolutionary relations of key efflux determinants. Several putative efflux pump genes have been identified through genome analysis in these other species, but few have been characterized. There are some studies on the impact of efflux on AMR in *S. epidermidis* (Huang et al., 2025; Ribič et al., 2020; Costa et al., 2018; García-Gómez et al., 2017; Marco et al., 2017), *S. haemolyticus* (Wang et al., 2021; Marco et al., 2017; Correa et al., 2008), and the coagulase-positive veterinary pathogen *Staphylococcus pseudintermedius* (Leal et al., 2023; Rampacci et al., 2022; 2025) but not for other staphylococci. Homologues of some *S. aureus* main MDR EP and other efflux pump genes have been identified through genome analysis, including *norB/C*, *sdrM* and *lmrS* in *S. epidermidis* (Floyd et al., 2010; Juárez-Verdayes et al., 2012; Costa et al., 2018), *mepA*, *lmrS* and *mdeA* in *S. haemolyticus* (Huang et al., 2004; Floyd et al., 2010; Magnan et al., 2024), a MDR EP gene of the MATE family in *S. lugdunensis* HKU09-01 (SLGD_00116) (Tse et al., 2010; Heilbronner et al., 2011) and *norB/C* in S*. haemolyticus*, *S. hominis*, *S. saprophyticus*, *S. capitis* and *S. carnosus* (Juárez-Verdayes et al., 2012), but most of these efflux systems were not characterized. Additional plasmid-encoded efflux pumps, such as QacG, QacH and QacJ have been described in *S. aureus* and other clinically relevant staphylococcal species (Correa et al., 2008; Wassenaar et al., 2015; Teixeira et al., 2023; Morais et al., 2025).

NorA is one of the most studied transporters that plays a central role in reduced susceptibility to fluoroquinolones and biocides (Kaatz et al., 1993; DeMarco et al., 2007; Huet et al., 2008; Costa et al., 2011; Furi et al., 2013), acting as a first-step resistance mechanism towards these antimicrobial agents (Costa et al., 2015; Papkou et al., 2020). Four different *norA* alleles have been described in *S. aureus* (Yoshida et al., 1990; Kaatz et al., 1993; Noguchi et al., 2004; Costa et al., 2019) and they were shown to be associated with specific clonal lineages; the most the prevalent *norAI* and *norAII* alleles, each associated with specific major lineages of *S. aureus*, such as clonal complex 5 (CC5), CC8 (*norAI*) and CC22 (*norAII*), *norAIII,* associated with the CC45 lineage and the *norA*_CC59-CC121_ associated with CC59 and CC121 lineages (Costa et al., 2019; Ferreira et al., 2022). While *norA* appears to be conserved across the genus (Ferreira et al., 2022), its genetic variability and the diversity of other major efflux determinants remains largely unexplored, highlighting the need for comparative and evolutionary studies across clinically relevant species.

Several goals were established for this study. First, we aimed to assess the presence and genetic variability of main efflux pump genes *norA*, *norB*, *norC* and *mepA* in *S. aureus* and other pathogenic staphylococcal species; *S. epidermidis*, *S. haemolyticus*, *S. hominis* and *S. lugdunensis*, by combining an *in silico* analysis of the MDR transporters in these five species with the genotypic characterization of *norA* in clinical strains, in order to gain further insight into the diversity and potential contribution of these determinants to efflux-mediated resistance. By further analyzing the data gathered, we then sought to characterize the evolution of the *norA* gene across these five species and to clarify potential phylogenetic relations between the *norB*, *norC*, *norB/C* and related genes. Finally, we aimed to identify additional putative chromosomally encoded MDR transporters in these five clinically relevant staphylococcal species, expanding the current knowledge of their MDR transporter repertoires.

## 2 Materials and methods

### 2.1 *In silico* analysis of main MDR EP genes variability and corresponding polypeptide sequences

Nucleotide sequences and corresponding amino acid sequences of *norA*, *norB, norC*, *norB/C* and related sequences (SACOL2449, SE2010, SE0196, SH0432, SH0614) and *mepA* genes from 100 *S. aureus*, 100 *S. epidermidis*, 44 *S. haemolyticus*, 26 *S. hominis* and 36 *S. lugdunensis* genomes available at RefSeq (O’Leary et al., 2016) until November 2025 were retrieved for analysis (Supplementary Tables S1–S5). An additional set of 10 *S. haemolyticus*, 7 *S. lugdunensis* and 5 *S. hominis* genomes sequenced at the chromosome level was included to increase the data analyzed. The genomes were selected based on the strain clonal lineages, when that information was available, to ensure diversity of the dataset. Duplicated genomes were excluded. The quality of each genome was assessed by evaluating the assembly continuity, completeness, total length, species match and absence of contaminations.

These five species were selected because of their clinical relevance (Pain et al., 2019; Becker et al., 2020; De Oliveira et al., 2020), and availability of sufficient genome sequences to support comparative genomic and phylogenetic analyses. The efflux pump genes *norA*, *norB*, *norC*, *norB/C* and *mepA* were selected as the main chromosomally encoded multidrug efflux pump genes shared among these species.

The analysis of *S. aureus norA*/NorA variability was performed with 46 sequences from strains with publicly available genomes described above and from additional MRSA clinical isolates previously studied and representative of main clonal lineages, corresponding to 21 NorAI, 12 NorAII, 4 NorAIII and 9 NorA_CC59–CC121_ (Costa et al., 2019; Ferreira et al., 2022).

For each species, nucleotide and amino acid sequences were aligned with the Muscle algorithm provided in MEGA v11 software package (Tamura et al., 2021) to assess gene variability and alterations in the corresponding polypeptide sequences. Allelic profiles of the *S. aureus*, *S. epidermidis, S. haemolyticus* and *S. hominis* sequences were retrieved from the PubMLST database (https://pubmlst.org/, accessed in January 2026) to establish relations between clonal lineages and gene diversity. This analysis was not performed for *S. lugdunensis*, as no multilocus sequence typing scheme is currently defined for this species in the database.

Transmembrane segments (TMS) and conserved motifs described for MFS transporters with 12 and 14 TMS (Paulsen et al., 1996; Kumar et al., 2021; Brawley et al., 2022; Shang et al., 2022), and for MATE family transporters (Begum et al., 2005) were identified and compared between sequences of each staphylococcal species. Key residues for NorA, NorB, NorC, NorB/C and MepA substrate binding and drug resistance were compared between sequences.

The *in silico* platform PHYRE^2^ (Protein Homology/analogY Recognition Engine v2.2) (Powell et al., 2025) was used to identify the tridimensional structure most similar to *S. aureus* NorAI, namely, the c7Lo8z model of NorA protein complexed with Fab36 (Brawley et al., 2022). Impact of possible residue substitutions on NorA activity was predicted with the SuSPect algorithm (Yates et al., 2014), producing a table of scores from 0 to 100 according to predicted deleteriousness effects (0 = neutral to 100 = deleterious). A score of 50 has been recommended as a cut-off between neutral and deleterious variants, with extreme scores allowing more confident predictions (Yates et al., 2014). A threshold of ≥75 was selected to prioritize high-confidence deleterious predictions.

### 2.2 Genotypic analysis of clinical strains

#### 2.2.1 Bacterial strains

A set of 99 clinical strains was included to complement the *in silico* analysis. This comprised 52 *S. epidermidis*, 23 *S. haemolyticus* and 24 *S. hominis* non-duplicate clinical isolates from several infection sources collected consecutively during 2023 in a hospital in Greater Lisbon, Portugal (Supplementary Tables S6–S8). Information about the clonal lineage was not available for the clinical isolates. Few *S. lugdunensis* clinical strains were available and thus were not included in this analysis. All isolates were grown in tryptic soy broth with shaking or tryptic soy agar at 37 °C. Both media were acquired from Oxoid™ (Hampshire, UK). Species identification was confirmed by amplification of the *nuc* gene following the protocol described by Hirotaki and colleagues (Hirotaki et al., 2011).

#### 2.2.2 Analysis of *norA* gene variability in clinical isolates

The *norA* gene of the 99 clinical isolates was amplified by PCR using two sets of species-specific primers to cover the entire gene and promoter regions (Supplementary Table S9).

PCR reaction mixtures were prepared in 0.025 mL containing 0.75 U of NZYTaq II; 1X Taq buffer; 0.4 μM of each primer; 0.2 mM dNTPs; 1.75 mM MgCl_2_. All PCR reagents were acquired from NZYTech (Lisbon, Portugal). The amplification conditions are described in Supplementary Table S10.

Amplification products were purified using the kit NZYGelpure (NZYTech) and sequenced at STAB VIDA (Caparica, Portugal). Sequences were analyzed using the programs SnapGene Viewer (GSL Biotech; available at snapgene.com) and blastx (NCBI, Bethesda, MD, United States). The *norA* gene sequences determined for the clinical isolates were deposited at the GenBank and the corresponding NCBI accession numbers are listed in Supplementary Tables S6–S8.

### 2.3 Phylogenetic analyses

Phylogenetic analyses were performed for the dataset described in Section 2.1, excluding identical sequences with Cd-hit (Li and Godzik, 2006), resulting in a final number of 88 representative sequences of the *norA* gene and a total of 77 representative sequences of the *norB*, *norC*, *norB/C* genes and related sequences.

The 88 *norA* sequences included 25 *S. aureus*, 25 *S. epidermidis*, 11 *S. haemolyticus*, 20 *S. hominis* and 7 *S. lugdunensis* sequences. The 25 *S. aureus norA* sequences included nine *norAI*, seven *norAII*, two *norAIII,* seven *norA*_*CC59-CC121*_ and at least one genome of the main clonal lineages (i) more frequently associated with infection and (ii) associated with each *norA* allele (Supplementary Table S1). The 25 *S. epidermidis norA* sequences also included at least one genome of the main clonal lineages more frequently associated with infection (Supplementary Table S2). Most *S. haemolyticus*, *S. hominis* and *S. lugdunensis norA* sequences analyzed included genomes for which no information about their clonal lineages was available.

The phylogenetic analysis of the *norB*, *norC*, *norB/C*, SACOL2449 and SE0196 genes included 13 *S. aureus norB*, 10 *S. aureus norC*, 37 *norB/C* [*S. epidermidis* (n = 10), *S. haemolyticus* (n = 10), *S. hominis* (n = 10) and *S. lugdunensis* (n = 7)], 15 SACOL2449 [*S. aureus* (n = 10) and *S. haemolyticus* (n = 5)], and two SE0196 (1 *S. epidermidis* and 1 *S. lugdunensis*) (Supplementary Tables S1–S5).

TREE PUZZLE v5.2 was used to evaluate the phylogenetic signal of the sequences (Schmidt et al., 2002). Codon-based tests of positive selection were performed using the methods SLAC and FEL available in the DATAMONKEY server (https://www.datamonkey.org/slac; https://www.datamonkey.org/fel/) (Weaver et al., 2018). Sequences were then aligned using the Muscle algorithm and exported to IQ-TREE v1.6.1 (Nguyen et al., 2015) for phylogenetic analysis. After selection of the best substitution model, a maximum likelihood tree was reconstructed using a GTR + F + I + G4 model, the ultra-fast bootstrapping option (Hoang et al., 2018), and approximate likelihood-ratio test support values (Guidon et al., 2010) calculated from 1,000 replicates. Visualization of the resulting phylogenetic tree was performed with FigTree v1.4.4 (https://tree.bio.ed.ac.uk/software/figtree/).

All comparative analyses were based on nucleotide sequences to allow characterization of allelic diversity, to assess genome changes resulting from selective pressures and evolutionary relations.

### 2.4 *In silico* identification of MDR transporters

Putative MDR transporters were searched for *S. aureus* COL, *S. epidermidis* ATCC 12228, *S. haemolyticus* JCSC1435 and *S. lugdunensis* HKU09-01 on the TransportDB 2.0 database (Elbourne et al., 2017; Elbourne et al., 2023) http://www.membranetransport.org/transportDB2/index.html (last access: November 2025) and the Transporter Classification Database (Saier et al., 2021) https://www.tcdb.org (last access: November 2025). The nucleotide sequences of each MDR transporter of *S. aureus* COL strain were used as queries for BLAST analysis against a subset of the genome dataset described in 2.1, for the sake of feasibility, consisting of *S. aureus* (n = 30), *S. epidermidis* (n = 30), *S. haemolyticus* (n = 41), *S. lugdunensis* (n = 32) and *S. hominis* (n = 22) (Supplementary Tables S1–S5). This analysis was performed using the blastn algorithm and inclusive criteria (expect value, E-value, threshold 0.05, nucleotide identity ≥60%, query coverage ≥70%). For BLAST results that did not meet the inclusive criteria (query coverage between 40% and 70%), the resulting amino acid sequences were aligned with the Muscle algorithm provided in MEGA v11 software package (Tamura et al., 2021), visualized with Jalview 2.11.3.1 (Waterhouse et al., 2009). and further analyzed with InterPro 97.0 (Paysan-Lafosse et al., 2023). *S. epidermidis*, *S. haemolyticus*, *S. lugdunensis* and *S. hominis* putative MDR transporters showing no significant nucleotide sequence similarity to any of the *S. aureus* COL MDR transporters were also BLAST searched against each of the other four species, including other *S. aureus* strains.

Additional candidate MDR EPs were searched based on the conserved motifs of transporter families with FIMO (Find Individual Motif Occurences, version 5.5.0) available at MEME suite (Grant et al., 2011; Bailey et al., 2015) (threshold p-value < 1E-4). In particular, motif A (GxLaDrxGrkxxI) motif B (lxxxRxxqGxgaa) and motif C (gxxxGPxxGGxl) of the MFS transporter superfamily and ABC superfamily Signature Motif (LSGGQ) were searched in the genomes of *S. aureus* COL, *S. epidermidis* ATCC 12228, *S. haemolyticus* JCSC1435 and *S. lugdunensis* HKU09-01. This analysis was not carried out for *S. hominis* as there were no complete genomes available in the FIMO database to perform the search. Instead, genes showing sequence similarity to those identified were searched by BLAST in *S. hominis* genomes.

### 2.5 Ethics statement

Ethical approval was not required for this study because it involved the analysis of bacterial cultures only and did not include human participants or the collection of identifiable personal data.

## 3 Results

### 3.1 Analysis of *norA* variability in *S. epidermidis, S. haemolyticus, S. hominis *and* S. lugdunensis*

The variability of the *norA* gene was assessed in published genomes and clinical isolates of *S. epidermidis* (n = 100 and n = 52, respectively), *S. haemolyticus* (n = 44 and n = 23, respectively), *S. hominis* (n = 26 and n = 24, respectively) and *S. lugdunensis* (n = 36, no data on clinical isolates). Overall, limited *norA* variability was observed in these species (Table 1). For *S. epidermidis*, the *norA* gene shared 100% sequence identity among all sequence type (ST) 2, ST5 and ST8 genomes and the most divergent *norA* sequence, from a ST1174 genome, shared 97.3% identity with those sequences. This observation extended to the 52 *S. epidermidis* clinical strains, for which the two most variable *norA* sequences presented 97.4% nucleotide identity with ST2/ST5/ST8 strains.

**TABLE 1:** Lowest percentage of identity of *norA* sequences in *Staphylococcus epidermidis*, *Staphylococcus haemolyticus*, *Staphylococcus hominis* and *Staphylococcus lugdunensis* from either published genomes or clinical strains.

Species	Published genomes	Clinical strains
*S. epidermidis*	97.3%	97.4%
*S. haemolyticus* ^a^	97.9%	99.8%
*S. hominis* ^a^	97.6%	97.4%
*S. lugdunensis*	99.1%	—

^a^ A *norA* pseudogene, resulting from of a frameshift was detected in 1 *Staphylococcus haemolyticus* genome (SCAID PHRX1-2019) and in 1 *Staphylococcus hominis* genome (FDAARGOS_661). These pseudogenes were not included in the analysis.

The most divergent *S. haemolyticus norA* sequences shared 97.9% and 99.8% nucleotide identity in published genomes and clinical isolates, respectively. In *S. hominis,* the most variable *norA* sequences shared 97.6% and 97.4% nucleotide identity in published genomes and clinical isolates, respectively. The *norA* gene of *S. lugdunensis* was the least variable, with the most divergent *norA* sequence sharing 99.1% nucleotide identity (Table 1).

### 3.2 Phylogenetic analysis of *norA* in *S. aureus, S. epidermidis, S. haemolyticus, S. hominis *and* S. lugdunensis*

To characterize the evolution of *norA* across the five staphylococcal species, a subset of 88 *norA* sequences, retrieved either from published genomes or clinical isolates data and representing all *norA* sequences and main clonal lineages, was aligned and subjected to phylogenetic analysis. The dataset used was characterized by a high phylogenetic signal, as revealed by a high percentage (96.1%) of totally resolved randomly sampled sequence quartets, defined by likelihood mapping.

Figure 1 shows the maximum likelihood phylogenetic tree reconstructed from these sequences. Analysis of the phylogenetic tree reveals a segregation of the different *norA* alleles within *S. aureus*, a pattern that is not observed in the other staphylococcal species analyzed. In *S. epidermidis*, *S. haemolyticus*, *S. hominis* and *S. lugdunensis*, *norA* sequences cluster without forming well-supported allele-specific clades. Within *S. aureus*, the *norA*_*CC59–CC121*_ allele is split into two well-supported branches, indicating substantial intra-allelic variability. Interestingly, CC121 *norA* variants are positioned closer to the remaining *norA* alleles. Also, a *S. aureus* genome belonging to ST45 was found to harbor the *norAII* allele (highlighted in purple in Figure 1), although this clonal lineage has previously been associated solely with the *norAIII* allele. The quality of this genome sequence (CP083259.1) was assessed to exclude contamination, assembly or annotation issues and its assignment to ST45 was also confirmed.

**FIGURE 1:**
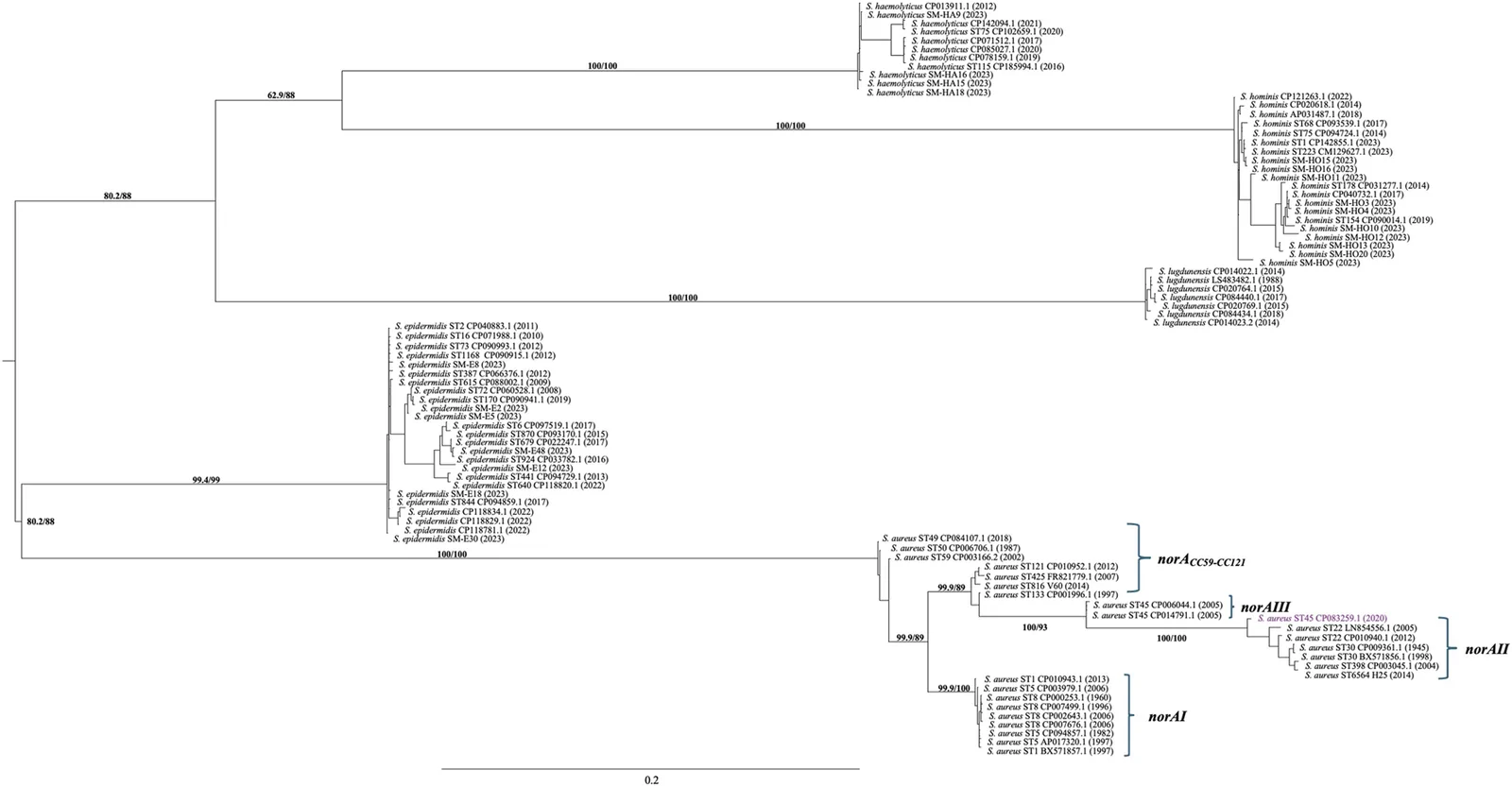
Phylogenetic analysis of the *norA* efflux pump genes of *Staphylococcus aureus*, *Staphylococcus epidermidis*, *Staphylococcus haemolyticus*, *Staphylococcus hominis* and *Staphylococcus lugdunensis*. Maximum likelihood consensus tree rooted at midpoint and drawn to scale, with branch lengths in the scale of nucleotide substitutions per site. Approximate likelihood-ratio test and bootstrap support values are illustrated at branch nodes, respectively. For each *norA* sequence, the species of origin, sequence type (when available), NCBI genome reference or clinical strain and year of isolation are indicated. The sequence highlighted in purple corresponds to the *Staphylococcus aureus* ST45 genome that carries the *norAII* allele.

### 3.3 Comparative analysis of NorA polypeptide sequences from different *S. aureus norA* alleles

The comparative analysis of *S. aureus* NorA sequences corresponding to the *norA* alleles known revealed several amino acid alterations, mostly located in the loops between transmembrane segments (TMS 3 and TMS 4, TMS 4 and TMS 5, TMS 6 and TMS 7, TMS9 and TMS10) and in transmembrane segments TMS 6, TMS 7 and TMS 9. Of these, we highlight the amino acid in the 119th position, which lies within motif-1, previously associated with NorA efflux capacity, and varies according to the different *norA* alleles; for NorAI, NorAIII and the NorA_CC59–CC121_ variant found in CC121 strains it corresponds to an isoleucine, that is replaced by a valine in NorAII and the NorA_CC59-CC121_ variant found in CC59 strains. Amino acid substitutions associated with each *S. aureus norA* allele were identified and are represented in Figure 2.

**FIGURE 2:**
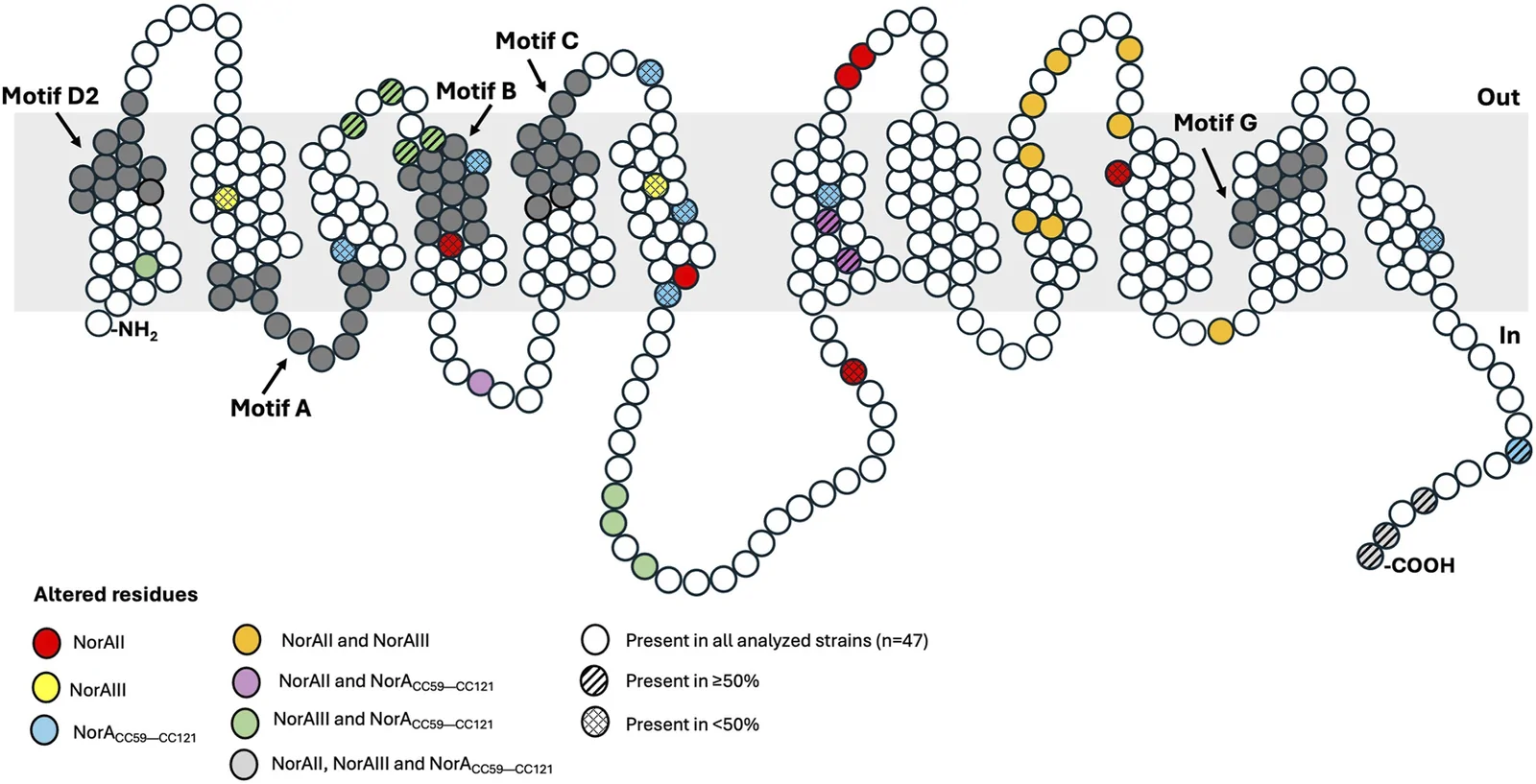
NorA predicted topology and substitutions associated with each *norA* allele in *Staphylococcus aureus*. The figure represents the NorAI sequence and altered residues on other alleles are represented by circles of different color: Red: altered in NorAII; Yellow: altered in NorAIII; Blue: altered in NorA_CC59–CC121_; Orange: altered in NorAII and NorAIII; Purple: altered in NorAII and NorA_CC59–CC121_; Green: altered in NorAIII and NorA_CC59–CC121_; Light grey: altered in NorAII, NorAIII and NorA_CC59–CC121_; No pattern circles: the substitutions are present in all analyzed sequences; Diagonal line circles: the substitutions are present in ≥50% but not all analyzed sequences; Crossed line circles: the substitutions are present in <50% of analyzed sequences. The conserved motifs D2, A, B, C and G are represented by the dark grey circles. This figure was adapted from Putman et al., 2000, taking into consideration the published structure of the NorA-Fab36 complex (PDB 7LO8) (Brawley et al., 2022).

### 3.4 Comparative analysis of NorA polypeptide sequences in five pathogenic staphylococcal species

The conserved motifs A, B, C, D2, G, 1 and 2 described for *S. aureus* NorA were identified in all the predicted NorA sequences of *S. epidermidis*, *S. haemolyticus*, *S. hominis* and *S. lugdunensis*. Alterations in the NorA polypeptide sequences were also analyzed, including in key residues for substrate binding and drug resistance based on what is known for *S. aureus* NorA. These residues were found to be conserved in the NorA sequences of the four remaining species. The only exceptions corresponded to one *in silico S. epidermidis* sequence (*S. epidermidis* PH1-28, ST387), which carried the substitution F306S, and 1 *S. epidermidis* clinical strain (SM-E8) which carried the substitution G143R in TM8.

Although no *norA* alleles were identified in *S. hominis*, the predicted NorA polypeptide sequences of this species were the most variable, differing by up to 2.6%, while the most variable NorA sequences of *S. epidermidis*, *S. haemolyticus* and *S. lugdunensis* differ only by 1.6%, 0.8%, and 0.5%, respectively. When compared to the NCBI reference sequence FDAARGOS_747, the most frequent substitution detected among the *S. hominis* clinical isolates and published genomes was Q380K (Table 2).

**TABLE 2:** NorA substitutions in *Staphylococcus hominis* clinical isolates and published genomes when compared with the reference genome FDAARGOS_747. SM-HO data refer to clinical strains; the remaining refer to published genomes.

Strain	Amino acid substitutions																			
	I121	A122	V153	I167	I168	A184	E185	K194	Q200	V213	A236	A250	M263	R264	T266	S349	V370	K378	Q380	R385
SM-HO2	K121	​	​	V167	M168	V184	​	​	​	​	​	​	​	​	I266	​	I370	N378	K380	G385
SM-HO3	K121	V122	​	V167	M168	V184	​	​	​	​	​	​	​	​	I266	​	I370	N378	K380	G385
SM-HO4	K121	V122	I153	V167	M168	V184	​	R194	​	​	​	​	​	​	I266	​	I370	N378	K380	G385
SM-HO5	​	​	​	V167	​	​	​	​	​	​	​	V250	​	K264	​	L349	​	​	K380	​
SM-HO10	K121	​	​	​	​	V184	​	​	​	​	​	​	​	​	​	​	I370	N378	K380	G385
SM-HO11	​	​	​	​	​	​	​	​	​	​	​	​	​	​	I266	​	​	N378	K380	G385
SM-HO12	K121	​	​	V167	M168	V184	​	​	​	​	​	​	​	​	​	​	​	​	​	​
SM-HO13	K121	​	​	V167	M168	V184	​	​	​	​	​	​	​	​	​	​	​	N378	K380	G385
SM-HO17	K121	​	​	V167	M168	V184	​	​	​	​	​	​	​	​	I266	​	I370	N378	K380	G385
SM-HO19	K121	​	​	V167	M168	V184	​	​	​	​	​	​	​	​	​	​	​	N378	K380	G385
SM-HO20	K121	​	​	V167	M168	V184	​	​	​	​	​	​	​	​	​	​	​	N378	K380	G385
19A	K121	​	​	V167	M168	​	​	​	​	​	​	​	​	​	​	​	​	N378	K380	G385
acrlk	​	​	​	V167	M168	​	​	​	​	​	​	​	​	​	I266	​	I370	N378	K380	G385
FDAARGOS 745	​	​	​	V167	M168	​	K185	​	​	​	T236	​	​	​	​	​	I370	N378	K380	G385
FDAARGOS 746	​	​	​	​	​	​	K185	​	E200	​	​	​	​	​	​	​	​	​	K380	​
FSEL1	​	​	​	​	​	​	K185	​	​	​	​	​	​	​	​	​	​	​	K380	​
K1	​	​	​	​	​	​	​	​	​	​	​	​	I263	​	​	​	​	​	K380	​
NT-2005	K121	​	​	​	​	​	​	​	​	​	​	​	​	​	​	​	​	​	​	G385
S34-1	K121	​	​	V167	M168	​	​	​	​	​	​	​	​	​	I266	​	I370	N378	K380	​
Wikim0113	K121	​	​	V167	M168	​	​	​	​	​	​	​	​	​	​	​	​	N378	K380	G385
YLP1	K121	​	​	V167	M168	​	​	​	​	L213	​	​	​	​	I266	​	I370	N378	​	G385

None of the alterations described above includes the main residues assigned for *S. aureus* NorA substrate binding and drug resistance (Brawley et al., 2022; Shang et al., 2022), which is in accordance with the apparent NorA functional conservation within these five staphylococcal species (Figure 3).

**FIGURE 3:**
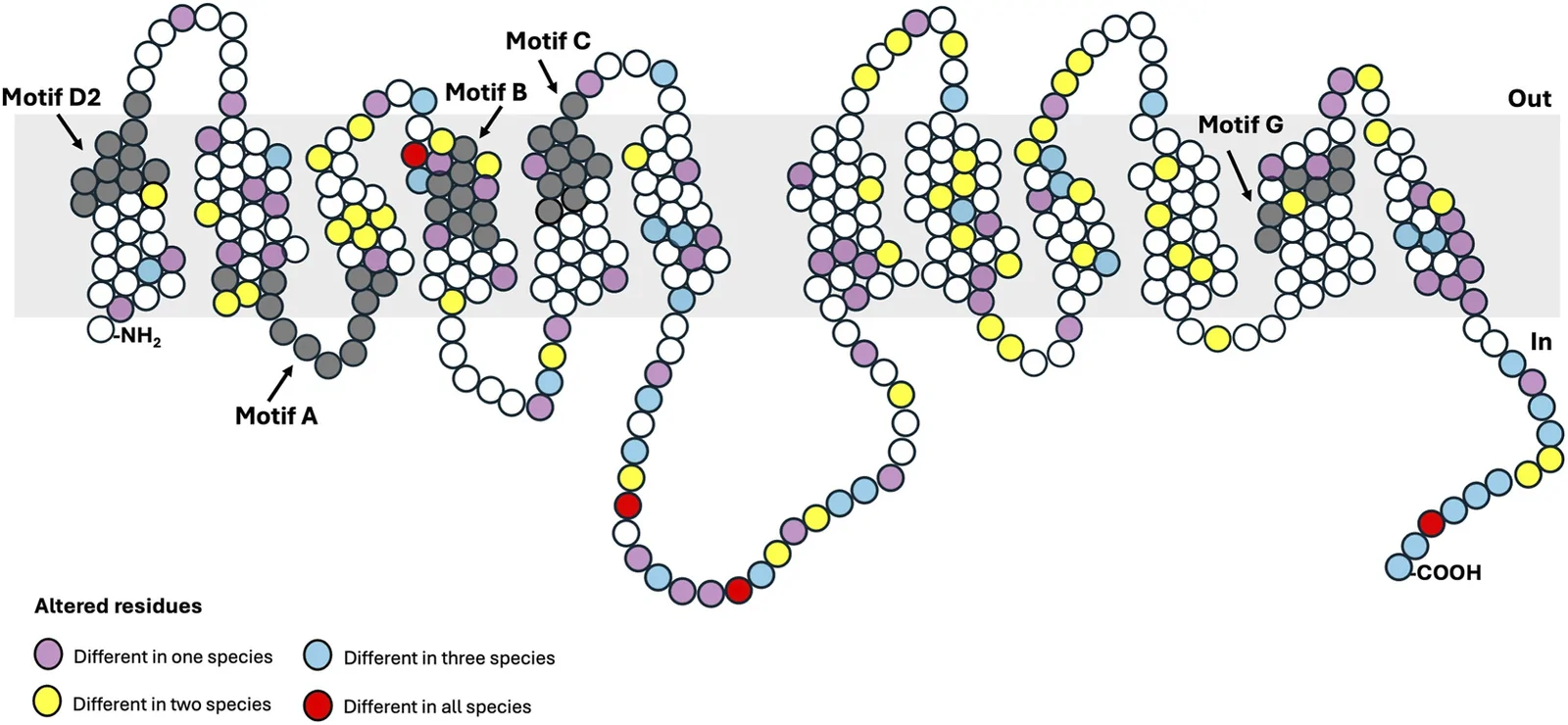
Comparison of predicted NorA topology and substitutions in *Staphylococcus aureus*, *Staphylococcus epidermidis*, *Staphylococcus haemolyticus*, *Staphylococcus lugdunensis* and *Staphylococcus hominis*. The figure represents the *Staphylococcus aureus* NorAI sequence and variable residues in other species, corresponding to substitutions present in ≥50% of the sequences of each analyzed species, are highlighted in different colors. Purple: the residue is altered in one species; yellow: the residue is altered in two species; blue: the residue is altered in three species; red: the residue is different in the five species analyzed. Conserved motifs D2, A, B, C and G are represented by the dark grey circles. This figure was adapted from Putman et al., 2000, taking into consideration the structure of the NorA-Fab36 complex (PDB 7LO8) (Brawley et al., 2022) and the analysis of 45 NorA sequences from 5 *Staphylococcus aureus* (1 NorAI, 1 NorAII, 1 NorAIII and 2 NorA_CC59–CC121_), 10 *Staphylococcus epidermidis*, 10 *Staphylococcus haemolyticus*, 10 *Staphylococcus lugdunensis* and 10 *Staphylococcus hominis*. These sequences are a subset of the dataset described in 2.1 and 2.2.1 and were chosen according to their NorA variability and clonal lineages, when available.

Next, we investigated the diversity of other major efflux pump genes.

### 3.5 Analysis of *norB*/NorB and *norC*/NorC variability in *S. aureus* published genomes

The presence of *norB* and *norC* genes and their variability was assessed in all *S. aureus* genomes included in this work, belonging to the main clonal lineages (Supplementary Table S1).

Two potential alleles were found for *S. aureus norB*: *norBI*, present in 93% (93/100) of the analyzed genomes and *norBII*, found only in CC45 genomes (7/100, 7%), and sharing 86.8%–87.9% sequence identity to *norBI*. These *norBII* sequences have a deletion of three nucleotides at the end of the gene, resulting in a NorBII protein with 462 amino acids, one less than NorBI. Among the *norBI* sequences, 37.5% (12/32) of the CC8 strains have a deletion of five nucleotides followed by an insertion of 14 nucleotides at the end of the gene, resulting in a protein with 466 amino acids, three more than the reference NorBI sequence (Figure 4). The impact of these alterations on NorB function is unknown. The complete alignment of the NorB sequences is presented in Supplementary Figure S1.

**FIGURE 4:**
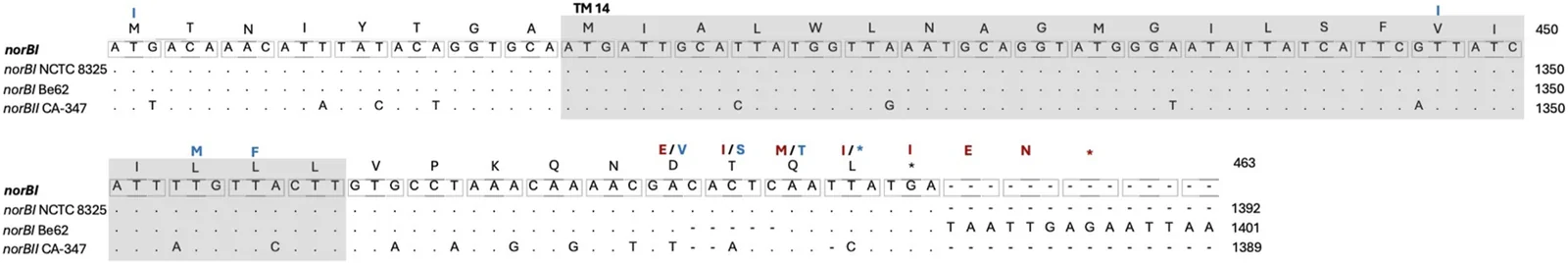
Partial multiple alignment of *Staphylococcus aureus norBI* and *norBII* sequences, highlighting differences between the *norB* alleles and variants at their C-terminal. The three sequences are representative of *norBI* with 463 (*Staphylococcus aureus* NCTC 8325) and 466 (*Staphylococcus aureus* Be62) amino acids, and of *norBII* (*Staphylococcus aureus* CA-347). The corresponding polypeptide sequence of *norBI* NCTC 8325 is shown above the alignment. The shaded regions correspond to the approximate positions of the transmembrane segments, predicted with DeepTMHMM 1.0 (Hallgren et al., 2022) and Phobius (Käll et al., 2007), using the NorBI sequence of *Staphylococcus aureus* NCTC 8325. The amino acid regions inside the boxes are relative to the conserved motifs described for MFS transporters with 14 TMS. The red and blue residues shown above the main polypeptide sequence represent the variations present in the polypeptide sequences of NorBI Be62 and NorBII CA-347, respectively, relatively to the NorBI of NCTC 8325.

The single CC50 genome analyzed (*S. aureus* 6850) did not carry the *norB* gene. Comparison of the regions downstream and upstream of the *norB* gene revealed a deletion of four genes in this genome, including *norB* and genes associated with virulence.

For *S. aureus norC,* no potential alleles were found. The two most divergent sequences belong to strains from the CC5 and CC22 lineages and share 96.7% nucleotide identity. At the protein level, these sequences differ by only 1.9%.

### 3.6 Phylogenetic analysis of *norB, norC, norB/C* and related sequences in published genomes

While *S. aureus* harbors two distinct transporters, NorB and NorC, which share approximately 70% identity at the nucleotide level (Kumar et al., 2021), *S. epidermidis*, *S. haemolyticus*, *S. hominis* and *S. lugdunensis* possess only NorB/C, previously described as a homologue of *S. aureus* NorC (Juárez-Verdayes et al., 2012). Additionally, for *S. epidermidis* and *S. haemolyticus*, two NorB/C proteins have been described (Juárez-Verdayes et al., 2012) but their relation with *S. aureus* NorB and NorC remains unclear. The corresponding genes in each species are described in Table 3 and their phylogenetic relations illustrated in Figure 5.

**TABLE 3:** Lowest percentage of identity of *norB, norC, norB/C or norB/C-like* sequences in *Staphylococcus aureus*, *Staphylococcus epidermidis*, *Staphylococcus haemolyticus*, *Staphylococcus hominis* and *Staphylococcus lugdunensis* published genomes.

Species	*norB*	*norC*	*norB/C*	*norB/C-like*
*S. aureus*	86.8%	96.7%	97.6%	—
*S. epidermidis*	—	—	97.7%^a^	—
*S. haemolyticus*	—	—	94.9%^b^ ^,^ ^c^	98.1%^e^
*S. hominis*	—	—	97.9%^d^	—
*S. lugdunensis*	​	—	99.6%	—

^a^ Two *Staphylococcus epidermidis* genomes (SCAID OTT1-2021 and sep6), carried a *norB/C* pseudogene as a result of a frameshift.

^b^ One *Staphylococcus haemolyticus* genome (46D_3) carried a *norB/C* pseudogene as a result of a frameshift.

^c^ Six *Staphylococcus haemolyticus* genomes (6/44, 13.6%) carried an ATT, insertion at the beginning of the gene resulting in a NorB/C-like with an additional methionine at the start.

^d^ One *Staphylococcus hominis* genome (FDAARGOS_661) carried a *norB/C* pseudogene as a result of a frameshift.

^e^ Eleven *Staphylococcus haemolyticus* genomes (11/44, 25%) did not have a *norB/C-like* gene.

**FIGURE 5:**
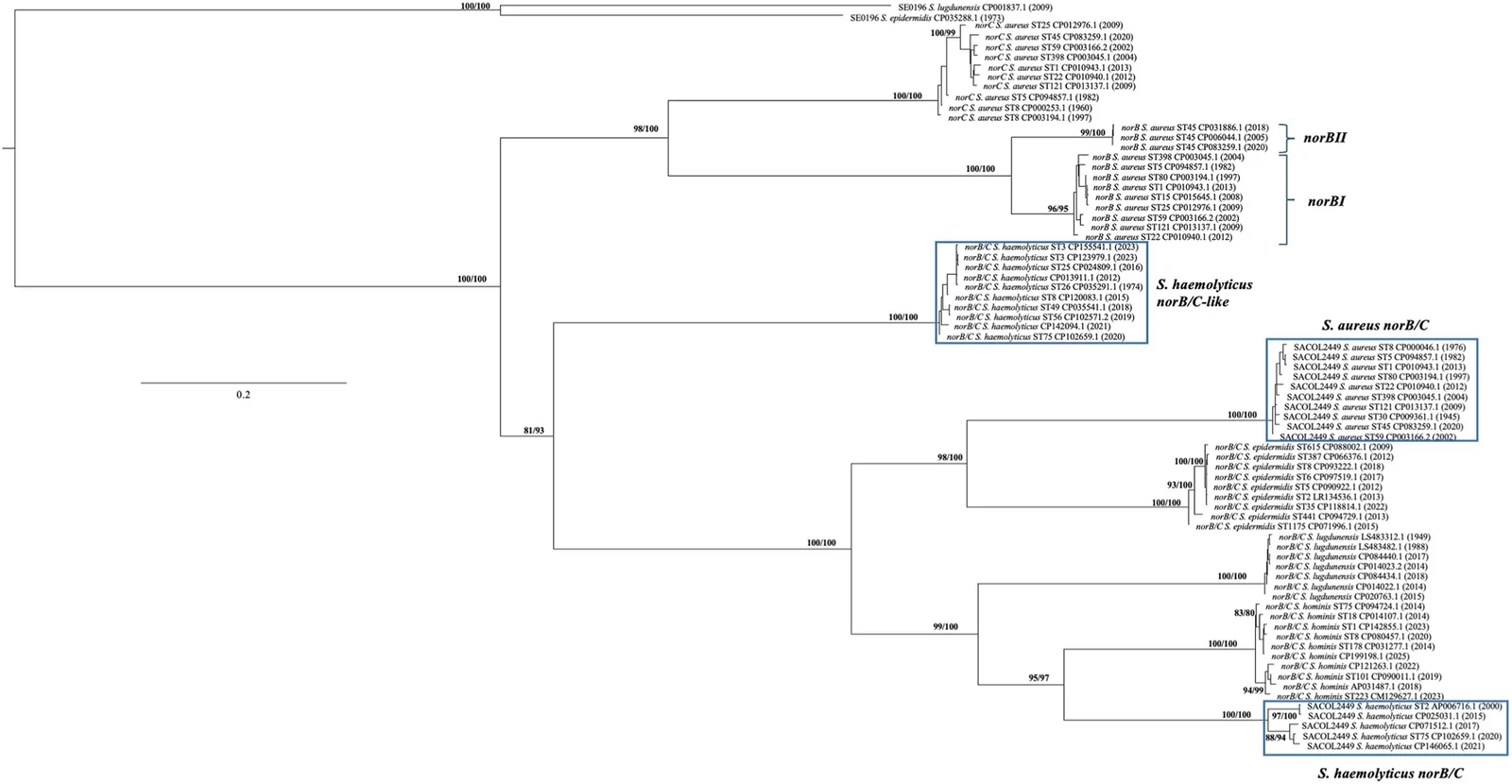
Phylogenetic analysis of the *norB*, *norC*, *norB/C*, *norB/C-like* and SE0196 efflux pump genes from *Staphylococcus aureus*, *Staphylococcus epidermidis*, *Staphylococcus haemolyticus*, *Staphylococcus hominis* and *Staphylococcus lugdunensis*. Maximum likelihood consensus tree rooted at midpoint and drawn to scale, with branch lengths in the scale of nucleotide substitutions per site. Approximate likelihood-ratio test and bootstrap support values are illustrated at branch nodes, respectively. For each gene sequence, the species of origin, sequence type (when available), NCBI genome reference and year of isolation are indicated. The boxes highlight the newly assigned *Staphylococcus aureus norB/C* and *Staphylococcus haemolyticus norB/C* and *norB/C*-like genes.

We observed that the *S. aureus* gene SACOL2449, previously described in the COL strain, displays approximately 67% nucleotide identity with both *S. aureus norB* and *norC,* suggesting a potential evolutionary relation between these genes. This gene was hereafter designated *S. aureus norB/C*.

The *S. epidermidis* SE2010 gene, according to its annotation in *S. epidermidis* ATCC 12228 strain, was hereafter designated *norB/C*. It shares approximately 66% nucleotide identity with a second *S. epidermidis* gene; SE0196 in ATCC 12228 strain. A putative homologue of SE0196 was also present in *S. lugdunensis*, but it was not detected in *S. aureus*, *S. haemolyticus* or *S. hominis*.

The *S. haemolyticus* gene corresponding to SH0614, according to its annotation in *S. haemolyticus* JCSC1435 strain was hereafter designated *norB/C*, while the second *S. haemolyticus* gene corresponding to SH0432 in *S. haemolyticus* JCSC1435 strain, was now designated *S. haemolyticus norB/C-like*. These genes also share approximately 66% nucleotide identity.

To clarify the evolutionary relations among the *norB*, *norC*, *norB/C, norB/C-like* and SE0196 genes, a subset of 77 nucleotide sequences from the five species in study was aligned and subjected to phylogenetic analysis. The dataset used was characterized by a high phylogenetic signal, as revealed by a high percentage (96.6%) of totally resolved randomly sampled sequence quartets, defined by likelihood mapping. Figure 5 shows the maximum likelihood phylogenetic tree reconstructed from these sequences.

Codon-based tests of positive selection were performed for every sequence showing no evidence of positive selection in any codon. Mutations under positive selection are favored as they provide a fitness advantage and can cause phylogenetic misinterpretations. Since none of the sequences analyzed in this work are under positive selection, the resulting phylogenetic tree should reflect the evolutionary relations between these genes.

The *S. aureus norB/C* gene is more closely related to the *norB/C* genes of *S. epidermidis*, *S. hominis*, *S. lugdunensis* and *S. haemolyticus,* rather than the *S. aureus norB* or *norC* genes. In contrast, the *S. epidermidis* SE0196 gene and its candidate homologue in *S. lugdunensis* form a distinct lineage, showing a distant relation with the *norB/C* genes.

The variability of the *norB/C* gene was assessed in the five species, whereas the variability of the *norB/C-like* gene was assessed in *S. haemolyticus*. Contrary to the segregation into different alleles described above for the *S. aureus norB* gene, no alleles were detected for the *norB/C* or *norB/C-like* gene of these species. *S. epidermidis norB/C* sequences from ST2, ST5 and ST8 genomes are almost identical, similarly to what was found for the *norA* sequences from these clonal lineages. Some of these genes were not present in all analyzed genomes of the same species. A summary of these findings is described in Table 3.

### 3.7 Analysis of *mepA*/MepA variability in published genomes

Putative homologues of *S. aureus mepA* were detected in *S. haemolyticus*, *S. lugdunensis* and in 2 *S. hominis* genomes (2/26, 7.7%), but were absent in *S. epidermidis*.

The variability of *mepA* was assessed and no potential *mepA* alleles were found. The most divergent *S. aureus* and *S. lugdunensis mepA* sequences share 97.4% and 99.5% identity, respectively. The 2 *S. hominis mepA* sequences were 99.9% identical. Although no *mepA* alleles were identified for *S. aureus*, we observed some association between clonal lineages and *mepA* gene variability. In particular, *mepA* from CC5 and CC8 *S. aureus* genomes share 99.5%–100% sequence identity while *mepA* from other CCs, including CC22 and CC121, are more variable (97.4%–98.5% sequence identity to *mepA* from CC5 and CC8 genomes).

Three *S. lugdunensis* genomes (DSM 4804, FDAARGOS_222 and SO-SLU20-1) carried a *mepA* pseudogene as a result of a frameshift and one genome (NCTC12217) had a 30-nucleotide deletion in this gene.

Only six out of the 44 *S. haemolyticus* genomes analyzed carried a complete *mepA,* with 99.3% sequence identity, and the corresponding protein is 452 amino acids long. The remaining had a truncated gene resulting from frameshifts or internal stop at different positions. The reference strain *S. haemolyticus* JCSC1435 carries two genes (SH0676 and SH0677), encoding proteins of 100 and 347 amino acids, respectively. Although these sequences show 100% nucleotide identity to portions of the functional *mepA* gene identified in other genomes, their predicted products are truncated and are therefore most likely not functional.

### 3.8 *In silico* identification of additional MDR transporters

Database search identified additional putative MDR transporters for *S. aureus* (30), *S. epidermidis* (35), *S. haemolyticus* (38) and *S. lugdunensis* (36). Most of these predicted transporters are classified within the MFS superfamily, followed by the ABC superfamily. No MDR transporters were detected for *S. hominis* in the Transporter Classification Database and there was no information for this species in the TransportDB 2.0 database.

Besides NorA and NorB/C, candidate homologues of *S. aureus* SdrM and SepA were also identified in the other four staphylococcal species studied. On the other hand, MdeA predicted homologues were only found in *S. haemolyticus*, *S. lugdunensis* and *S. hominis* while LmrS putative homologues were only found in *S. epidermidis*, *S. haemolyticus* and *S. lugdunensis*. Some MDR transporters were not present in all genomes of the same species. Several MDR transporters were also identified in *S. hominis,* for which there was no information in the databases.

Additional putative MDR transporters were identified with FIMO. This approach allowed the identification of one new candidate MDR transporter gene from the MFS superfamily for *S. aureus* (SACOL0261) which shares high sequence similarity with another putative MDR transporter determinants previously identified in the database for *S. haemolyticus* (SH0296) and *S. lugdunensis* (SLGD_00282). Several new candidate MDR transporter genes from the ABC superfamily were identified for *S. epidermidis* (SE0117, SE0198), *S. haemolyticus* (SH1095, SH1589) and S*. lugdunensis* (SLGD_00025, SLGD_00193, SLGD_00808, SLGD_01579, SLGD_01920, SLGD_01921, SLGD_02454, SLGD_02455). BLAST search was performed for each gene against the complete genomes of the other four staphylococcal species, and genes showing significant sequence similarity were found, including *S. epidermidis* ATCC12228 SE0988 and SE1550, *S. aureus* COL SACOL0264, SACOL1352, SACOL1924, SACOL2430, SACOL2431. FIMO search was not carried out for the MATE family since there is no conserved consensus sequence for this family of transporters. TransportDB search identified one putative MDR MATE transporter for *S. epidermidis* (SE1103), *S. haemolyticus* (SH0676) and *S. lugdunensis* (SLGD_00116). One candidate MDR transporter of the MOP superfamily was identified for *S. lugdunensis* (SLGD_00332).

The complete list of established and putative MDR transporter genes of *S. aureus* (n = 47), *S. epidermidis* (n = 44), *S. haemolyticus* (n = 43), *S. lugdunensis* (n = 49) and *S. hominis* (n = 32) is described in Table 4.

**TABLE 4:** List of established (white shaded cells) and putative (gray shaded cells) multidrug transporter genes in the five species studied.

Family	*S. aureus*	*S. epidermidis*	*S. haemolyticus*	*S. hominis*	*S. lugdunensis*
MFS	**SACOL0754 (*norA*)**	**✓**	**✓**	**✓**	**✓**
MFS	SACOL1475 (*norB*)	**X**	**X**	**X**	**X**
MFS	SACOL0086 (*norC*)	**X**	**X**	**X**	**X**
MFS	SACOL0070^a^	**X**	**X**	**X**	**X**
MFS	SACOL0103	**X**	**X**	**X**	**X**
MFS	SACOL0122 (*tet(38)*)	**X**	**X**	**X**	**X**
MFS	SACOL0163	**✓**	**X**	**X**	**X**
MFS	SACOL0200	**✓** ^e^	**X**	**X**	**X**
MFS	SACOL0620	**✓** ^f^	**✓** ^i^	**X**	**✓**
MFS	**SACOL0733**	**✓**	**✓**	**✓**	**✓**
MFS	SACOL0983	**X**	**X**	**X**	**X**
MFS	**SACOL1809**	**✓**	**✓**	**✓**	**✓**
MFS	SACOL2157 (*lmrS*)	**✓**	**✓**	**X**	**✓**
MFS	**SACOL2159 (*sdrM*)**	**✓**	**✓**	**✓**	**✓**
MFS	**SACOL2257**	**✓**	**✓**	**✓**	**✓**
MFS	**SACOL2347**	**✓**	**✓**	**✓**	**✓**
MFS	**SACOL2350**	**✓**	**✓**	**✓**	**✓**
MFS	SACOL2413 (*mdeA*)	**X**	**✓**	**✓**	**✓**
MFS	**SACOL2437**	**✓** ^g^	**✓**	**✓**	**✓**
MFS	**SACOL2449 (*norB/C*)**	**✓**	**✓**	**✓**	**✓**
MFS	**SACOL2460**	**✓**	**✓**	**✓**	**✓**
MFS	SACOL2471	**✓**	**X**	**X**	**✓**
MFS	SACOL2504^b^	**X**	**X**	**X**	**X**
MFS	SACOL2523^c^	**X**	**X**	**X**	**✓** ^m^
MATE	SACOL0405 (*mepA*)	**X**	**✓** ^j^	**✓** ^k^	**✓**
RND	**SACOL2252**	**✓**	**✓**	**✓**	**✓**
RND	**SACOL2566**	**✓** ^h^	**✓**	**✓** ^l^	**✓**
ABC	**SACOL0700**	**✓**	**✓**	**✓**	**✓**
ABC	**SACOL1353** ^d^	**✓**	**✓**	**✓**	**✓**
ABC	**SACOL1892**	**✓**	**✓**	**✓**	**✓**
MFS	SACOL0261	**X**	**✓**	**X**	**✓**
MFS	*nms*	**✓**	**X**	**X**	**X**
ABC	SACOL0264	**✓**	**X**	**X**	**✓**
ABC	SACOL1352	**✓**	**✓**	**X**	**✓**
ABC	SACOL1924	**✓**	**✓**	**X**	**✓**
ABC	SACOL2430	**X**	**X**	**X**	**✓**
ABC	SACOL2431	**X**	**X**	**X**	**✓**
SMR	**SACOL2158 (*sepA*)**	**✓**	**✓**	**✓**	**✓**
MFS	**X**	SE0196	**X**	**X**	**✓**
MFS	**✓**	SE0239	**✓**	**✓**	**X**
MFS	**X**	SE0241	**✓**	**X**	**✓**
MFS	**X**	SE0247	**✓**	**✓**	**✓**
MFS	**X**	SE0700	**X**	**X**	**✓**
MFS	**X**	SE2238	**✓**	**✓**	**✓**
MFS	**X**	SE2259	**✓**	**✓**	**X**
MATE	**X**	SE1103	**X**	**X**	**X**
SMR	**X**	SE1877	**X**	**✓**	**X**
SMR	**X**	SE1878	**X**	**✓**	**✓**
ABC	**X**	SE0095	**✓**	**✓**	**X**
ABC	**X**	SE0117	**X**	**X**	**✓**
ABC	**✓**	**SE0140**	**✓**	**✓**	**✓**
ABC	**X**	SE0994	**X**	**X**	**X**
MFS	**X**	**X**	SH0432 (*norB/C* -like)	**X**	**X**
MFS	**✓**	**✓**	SH0045	**X**	**X**
MFS	**✓**	**✓**	SH0152	**X**	**X**
MFS	**X**	**X**	SH0155	**X**	**X**
MFS	**✓**	**X**	SH0296	**X**	**✓**
MFS	**X**	**X**	SH0298	**✓**	**X**
MFS	**X**	**X**	SH1194	**X**	**X**
MFS	**X**	**X**	SH2649	**X**	**X**
MATE	**✓**	**X**	SH0676	**✓**	**✓**
SMR	**X**	**X**	SH0753	**✓**	**X**
SMR	**X**	**✓**	SH0754	**✓**	**X**
ABC	**✓**	**✓**	SH2614	**X**	**✓**
MFS	**✓**	**X**	**X**	**✓**	SLGD_00418
MFS	**X**	**✓**	**X**	**X**	SLGD_00646
MFS	**✓**	**X**	**X**	**X**	SLGD_02041
MFS	**X**	**X**	**X**	**X**	SLGD_02517
MATE	**X**	**X**	**X**	**X**	SLGD_00116
MOP	**X**	**X**	**✓**	**✓**	SLGD_00332
SMR	**X**	**X**	**X**	**X**	SLGD_00726
RND	**X**	**X**	**X**	**X**	SLGD_00168
ABC	**X**	**X**	**X**	**X**	SLGD_00808
ABC	**X**	**X**	**X**	**X**	SLGD_02455

(a, b, c, d) Genes present in 30.0% (9/30), 86.7% (26/30), 63.3% (19/30) and 93.3% (28/30) of the *Staphylococcus aureus* genomes, respectively; (e, f, g, h) genes present in 93.3% (28/30), 10% (3/30), 96.7% (29/30) and 60% (18/30) of the *Staphylococcus epidermidis* genomes, respectively; (i) gene present in 85.4% (35/41) of the *Staphylococcus haemolyticus* genomes; (j) 85.3% (35/41) of the *Staphylococcus haemolyticus* genomes carry a truncated gene (k, l) genes present in 9.1% (2/22) and 95.5% (21/22) of the *Staphylococcus hominis* genomes, respectively; (m) gene present in 56.3% (18/32) of the *Staphylococcus lugdunensis* genomes; Genes highlighted in bold: present in all five species; Check marks highlighted in green: present in ≥75% of the genomes; Check marks highlighted in grey: present in <75% of the genomes. The gene frequency analysis was only carried out for the first 30 genes (SACOL0754 (*norA*) to SACOL1892). Grey shading highlights newly identified putative transporters for each species, predicted through database search and sequence similarity.

Seventeen genes are common to the five species studied, including the main MDR transporter determinants *norA*, *norB/C*, *sdrM* and *sepA*. On the other hand, some genes were only found in one of the species (Table 4).

## 4 Discussion

This study investigated the genetic diversity, evolution and phylogenetic relations of efflux pump genes and other potential multidrug-resistance transporters in five clinically important staphylococcal species.

Among the five staphylococcal species analyzed in this work, only *S. aureus* exhibited detectable *norA* allelic diversity. The population structure of *S. aureus* is known to be highly clonal, with some dominant clonal complexes that have disseminated worldwide (Enright et al., 2002; Feil et al., 2003; Lee et al., 2018). In this species, evolutionary diversification has been driven predominantly by point mutations and bacteriophage-mediated events, rather than by homologous recombination, which contrasts with what is observed in *S. epidermidis*, for which frequent recombination contributes to a more heterogeneous population structure (Miragaia et al., 2008). Despite this higher genomic diversity, the *norA* gene in *S. epidermidis* displays lower sequence variability than in *S. aureus*, suggesting that differences in population structure alone do not account for the observed *norA* allelic diversification. An alternative explanation may lie in the phylogenetic distance separating the *norA* gene of *S. aureus* from that of the other four staphylococcal species included in this study (Ferreira et al., 2022).

The phylogenetic relations of the *norA* gene (Figure 1) are consistent with the results obtained in our previous study (Ferreira et al., 2022) and with the established evolutionary relations among staphylococci (Madhaiyan et al., 2020), reinforcing the presence of *norA* at an early stage in the evolutionary diversification of the *Staphylococcus* genus. *S. aureus* shows higher *norA* gene variation, with distinct *norA* alleles segregating within the species.

While each *S. aureus norA* allele forms a single branch, the *norA*_*CC59–CC121*_ allele is split into two well-supported branches, indicating substantial intra-allelic variability. In earlier work, a nucleotide identity cut-off of 95% was established to discriminate between different *norA* alleles. Although sequences assigned to the *norA*_*CC59–CC121*_ allele are the most variable, their nucleotide identity lies within this threshold. Nevertheless, the consistent separation into two phylogenetic branches suggests that the current classification criteria may underestimate the diversity of this allele. The limited representation of CC121 genomes in databases may influence the assignment of CC59 and CC121 *norA* variants within the same allele. Analysis of additional genome sequences shall clarify whether these sequences represent or not a single *norA* allele and their positioning compared to the other *S. aureus norA* alleles.

Previous studies from our group have shown that, in *S. aureus*, *norA* alleles are strongly associated with specific clonal lineages, with all strains from a given sequence type carrying the same *norA* allele (Costa et al., 2019; Ferreira et al., 2022). In the present study, an exception to this general pattern was identified. An ST45 *S. aureus* genome harbored the *norAII* allele, instead of the *norAIII* previously associated with this lineage. Although horizontal gene transfer could explain this result, *norA* is a core gene chromosomally-encoded, with no evidence of surrounding mobile genetic elements or phages, making this hypothesis highly unlikely. Given that *norAII* and *norAIII* are closely related yet form distinct phylogenetic clusters, this could indicate an ongoing diversification process within the ST45 lineage. This challenges the previously assumed strict correspondence between *norA* alleles and clonal lineages, which may require revision as more genomic data becomes available.

MFS transporters with 12 TMS, such as NorA, have five conserved motifs (A, B, C, D2, and G) (Paulsen et al., 1996). Two additional motifs were recently described for NorA based on a *S. aureus* NorAII sequence: motif-1 and motif-2 (Shang et al., 2022). Previous studies have identified several conserved residues that are key for NorA mediated drug resistance in *S. aureus,* including residues essential for efflux activity (Shang et al., 2021; 2022; Brawley et al., 2022). The comparative analysis of *S. aureus* NorA sequences from different *norA* alleles revealed no alterations in these residues, except the 119th residue, which is part of motif-1. The I119V substitution in *S. aureus* NorAII has been linked to increased efflux activity and decreased susceptibility to efflux pump substrates, including ciprofloxacin (Shang et al., 2022). We have now analyzed the impact of the I119V substitution using the SuSPect algorithm with the *S. aureus* NorA structural model described by Brawley and colleagues (Brawley et al., 2022) and no deleterious impact on the protein activity was predicted for this alteration.

The high *norA* sequence identity in the four CoNS species indicates limited genetic variability, suggesting strong evolutionary conservation of *norA*. Based on these preliminary results, there seem to be no *norA* alleles for these species. The confident assessment of allelic diversity depends on allele prevalence and sample size. While the analysis of 50–100 genomes may be sufficient to detect common alleles, 300 to 400 genomes may be required to robustly identify low-frequency variants (1% prevalence) and to exclude their presence with high confidence (Crossa, 1989). Therefore, the still limited number of genomes available may have restricted the detection of rare alleles for *S. haemolyticus*, *S. lugdunensis* and *S. hominis*. On the other hand, the data obtained with the clinical strains studied reflect the intraspecies *norA* variability obtained with *in silico* analysis. In particular, higher aminoacid variability was predicted for *S. hominis*, both from *in silico* genomes and clinical strains. The analysis of these *S. hominis* variants with SuSPect and the *S. aureus* NorA structural model (Brawley et al., 2022), predicted no impact on key residues, although the lack of a species-specific model may limit the accuracy of this analysis.

Similar to what has been described for *S. aureus*, an association between *norA* gene variability and clonal lineage also appears to occur in *S. epidermidis*. In contrast, no clear association was identified between clonal lineages and *norA* variability in *S. haemolyticus* and *S. hominis*. Only a limited number of genomes shared the same ST, which constrained the robustness of this analysis. Because clonal lineage information was not available for the *S. lugdunensis* genomes analyzed, this analysis was not performed for this species.

The variability of the *norB* gene was also associated with *S. aureus* clonal lineages in this study, that included all main *S. aureus* clonal lineages. Although the proposed *norBII* allele was only found in CC45 strains, it might also be present in other less represented clonal lineages not included in this work. Studies have reported that *S. aureus* CC45 occurs in both nasal colonization and bloodstream infections (Sangvik et al., 2011; Blomfeldt et al., 2013; Rasmussen et al., 2014; Roe et al., 2020; Nowrouzian et al., 2023). Previous studies from our group comprised only three CC45 isolates, among all the collections and datasets studied, that included over 1,000 genomes (Costa et al., 2019; Ferreira et al., 2022). The *norBII* allele shows up to 13.2% and 8.8% differences in the nucleotide and polypeptide sequences, respectively, compared to the more prevalent *norBI*. These occur mostly between amino acids 193 and 255, which spans the region between TMS 6 and TMS 7 through TMS 8 (Supplementary Figure S1). Previous studies have suggested that TMS 7 and 8 might influence substrate interactions among NorC-like transporters (Kumar et al., 2021). Given the similarity between NorC and NorB, we can speculate if the differences found in NorBII might have an impact on its substrate recognition and efflux activity. This variability may also impact on the design of specific efflux inhibitors.

For *S. aureus norC,* no potential alleles were found. The *S. aureus* NorC structure was already determined, and several conserved residues described as important for its tridimensional structure and interaction with substrate (Kumar et al., 2021). The few NorC alterations detected in the 100 *S. aureus* genomes analyzed do not affect any of these residues.

Due to the nucleotide similarity between *S. aureus norB* and *norC*, and the existence of a third related gene, *S. aureus norB/C* (previously described as SACOL2449), the evolutionary relations among these genes and their homologues in other staphylococcal species have remained unclear. As a result, previous studies have referred to *norB/C* carried by other staphylococcal species as a homologue of either *S. aureus norB* or *norC*. Additionally, two *norB/C* genes had been described for *S. epidermidis* and *S. haemolyticus*. To clarify these questions, we explored putative relations among these different genes in the five species in study, by a phylogenetic approach. The proposed annotation of SACOL2449 as *S. aureus norB/C* and SH0432 as *S. haemolyticus norB/C-like* was primarily supported by their phylogenetic clustering with *norB/C* genes from other staphylococcal species (Figure 5), together with their conserved motifs and sequence similarity to these homologues. In particular, SACOL2449 clustered closely with the *norB/C* genes of *S. epidermidis*, *S. hominis*, *S. lugdunensis* and *S. haemolyticus,* supporting its assignment as *S. aureus norB/C*. Similarly, *S. haemolyticus* SH0432 formed a distinct branch related to the *norB/C* group, supporting its designation as a *norB/C-like* determinant. These proposed annotations also aim to provide a more consistent nomenclature framework, as current database annotations frequently classify the *norB/C* and *norB/C-like* genes simply as *S. aureus norB* or *norC* homologues.

These findings raise the possibility that *S. aureus norB/C* may also play an important role in efflux response to antimicrobial exposure, similarly to what has been demonstrated for *norB/C* in *S. epidermidis* (Costa et al., 2018). To date, *S. aureus* NorB/C (SACOL2449) has only been described in two studies, as being potentially involved in biofilm formation (Scherr et al., 2013) and/or as putative quinolone and MDR transporter (Schindler et al., 2015).

Although 2 *S. haemolyticus norB/C* sequences shared 94.98% nucleotide identity (just below the 95% cut-off for new allele assignment), they were considered the same allele due to their close relation in the phylogenetic tree (Figure 5), which contrasts with the results for *S. aureus norBI* and *norBII*.

The phylogenetic analysis also suggests that *S. aureus norB* and *norC* as well as *S. haemolyticus norB/C-like* correspond to additional efflux pump genes that are absent in *S. epidermidis*, *S. lugdunensis* and *S. hominis.* These findings suggest distinct evolutionary trajectories of the *nor* efflux pump determinants among these staphylococcal species, with additional gene diversification in *S. aureus* and *S. haemolyticus*.

The *S. aureus* NorC structure (Kumar et al., 2021) can serve as a model for NorB and other transporters of the drug:H+ antiporters 2 family. The conserved motifs described for 14 TMS MFS transporters (A, B, C, D1, E, F, H) were screened in *S. aureus* NorB and NorC and in the NorB/C related sequences of the remaining species. All motifs, except C and D1, were present in the NorB, NorC and NorB/C and NorB/C-like predicted sequences of the five staphylococcal species studied. Most of NorC conserved residues with potential roles in its tridimensional structure and substrate interaction were also conserved in the NorB/C and NorB/C-like predicted sequences of each species.

Two conserved motifs have been described for some MATE family proteins (NiiLDpIFI and GAAiATvia) (Begum et al., 2005). These two motifs were present in all the *S. aureus*, *S. haemolyticus* and *S. lugdunensis* putative MepA sequences. A model of the MepA structure has been determined (Schindler et al., 2013), followed by the description of mutations associated with increased efflux (A302S, A392V, and A397V) or tigecycline resistance (L441W, T29I, E287G) (Schindler et al., 2013; Haim et al., 2017; Huang et al., 2023). The substitution A397V was detected in all CC30, CC121, CC133, CC398 and CC425 *S. aureus* genomes as well as in two of the CC22 genomes analyzed (2/5, 40%).

While previous studies have demonstrated that *norA* is part of the staphylococcal core genome (Costa et al., 2019; Ferreira et al., 2022), this analysis has not been carried out so far for the remaining efflux pump determinants. In this study, *norB* and *norC* were detected in all *S. aureus* genomes analyzed, while *norB/C* was identified in all *S. aureus*, *S. epidermidis, S. haemolyticus, S. lugdunensis* and *S. hominis* genomes, and *mepA* was present in all *S. aureus* and *S. lugdunensis* sequences (Table 4), suggesting that these genes may be part of each species core genome. Conversely, other genes were not found in all strains of the same species (Table 4). For instance, 25% of the *S. haemolyticus* genomes did not carry the *norB/C-like* gene. Only 2 *S. hominis* genomes (7.7%) carried *mepA,* while most of the *mepA* sequences carried by all *S. haemolyticus* corresponded to a truncated copy.

Seventeen out of the seventy-four total transporter genes analyzed in this work (23%) are common to the five species studied, including the main MDR transporter genes *norA*, *norB/C*, *sdrM* and *sepA*. *In silico* analysis identified several new putative MDR transporter genes for *S. aureus* (n = 18), *S. epidermidis* (n = 25), *S. haemolyticus* (n = 37), *S. lugdunensis* (n = 47) and *S. hominis* (n = 30). The polypeptide sequences of these candidate transporters were analyzed with InterPro for BLAST results that did not meet the inclusive criteria (query coverage <70%), namely, the SACOL0163 putative homologue in *S. epidermidis,* the SACOL1892 predicted homologues in *S. haemolyticus* and *S. lugdunensis* and the SACOL2566 putative homologue in *S. haemolyticus* (Supplementary Figure S2). These proteins share over 60% identity with their *S. aureus* counterparts, supporting a common ancestry, while differences in coverage and domain composition suggest possible structural and functional divergence.

This work presents some experimental and methodological limitations that should be acknowledged. One limitation concerns the representativeness of the clinical isolates collection. The strains used to analyze the variability of *norA* may be biased toward the most prevalent clonal lineages circulating in clinical settings, particularly since the isolates were recovered from the same hospital during the same year, potentially influencing the diversity observed in the *norA* gene. To mitigate this limitation, a large number of publicly available genomes representing diverse clonal lineages were included whenever possible. Another limitation worth noting is that most of the analyses presented were performed exclusively *in silico*, requiring future experimental functional validation. In particular, the putative MDR transporters identified across the five species require functional characterization to confirm their potential MDR transporter activity and to define their substrate profiles. These aspects should be explored in future studies to establish the biological and clinical relevance of the genetic variability described here and to further elucidate the contribution of these transporters to antimicrobial resistance in staphylococci.

## 5 Conclusion

To the best of our knowledge, this is the first study to analyze the variability of main efflux pump genes *norA*, *norB*, *norC*, *norB/C* and *mepA,* and the occurrence of several additional putative MDR transporters in five staphylococcal species of clinical relevance. Although the number of analyzed genomes for *S. haemolyticus, S. lugdunensis* and *S. hominis* may be a limitation of this study, it is a first step for further extended research on staphylococcal MDR transporters.

The results described reinforce *norA* as part of the staphylococcal genomic patrimony that follows the evolutionary pathway of these bacteria. Among the five staphylococcal species analyzed, only *S. aureus* showed *norA* allelic diversity. Two *norB* alleles were also described in *S. aureus* and associated with specific clonal lineages. Comparative analysis of NorB polypeptide sequences revealed differences between NorBI and NorBII that may influence substrate recognition or transport efficiency, although their functional impact remains unknown. Phylogenetic analysis revealed the existence of a *norB/C* gene in *S. aureus*, which is homologous to the *norB/C* genes of *S. epidermidis*, *S. haemolyticus*, *S. lugdunensis* and *S. hominis* and that *S. aureus norB* and *norC* as well as the *S. haemolyticus norB/C-like* gene may be related efflux pump genes that are absent in the other three species. By clarifying the evolutionary relations among these genes and suggesting an updated nomenclature accordingly, our analysis provides a framework for a more consistent annotation of these efflux pump determinants in *Staphylococcus.* This work has also identified several new putative MDR transporters in *S. aureus*, *S. epidermidis*, *S. haemolyticus*, *S. lugdunensis* and *S. hominis,* including candidate homologues to main MDR efflux pumps previously characterized in *S. aureus*.

The identification of additional putative MDR transporters further expands the known efflux repertoire in staphylococci and highlights the complexity of efflux-mediated resistance mechanisms. These results contribute to a better understanding of the genetic basis and evolution of efflux systems and their potential role in antimicrobial resistance. Future work should focus on the functional validation of these systems and their contribution to resistance phenotypes, which may ultimately support the development of novel therapeutic strategies targeting efflux-mediated resistance in staphylococci.

## Data Availability

Information for existing publicly accessible datasets is contained within the article. The datasets presented in this study can be found in online repositories. The names of the repositories and accession number(s) can be found in the Supplementary Material.
