# Supplementary material for: Occurrence, variability and evolutionary relations of efflux pump determinants across five pathogenic Staphylococcus species
Source: Front Pharmacol. 2026 Sep 18;17:1870078. doi: 10.3389/fphar.2026.1870078 (PMC13630626; doi:10.3389/fphar.2026.1870078)
Supplement: Supplementary file 1 [file Supplementaryfile1.docx]

Occurrence, variability and evolutionary relations of efflux pump determinants across five pathogenic *Staphylococcus* species

Carolina Ferreira^1^, Diana Martinho^1^, Ricardo Parreira^1^, José Melo-Cristino^2^, Miguel Viveiros^1^, Sofia Santos Costa^1^, Isabel Couto^1*^

^1^Global Health and Tropical Medicine, GHTM, LA-REAL, Instituto de Higiene e Medicina Tropical, IHMT, Universidade NOVA de Lisboa, Lisbon, Portugal

^2^Laboratório de Microbiologia, Serviço de Patologia Clínica, Centro Hospitalar Universitário Lisboa Norte, Lisboa, Portugal; Instituto de Microbiologia, Faculdade de Medicina, Universidade de Lisboa, Lisbon, Portugal

*** Correspondence:**
Isabel Couto
icouto@ihmt.unl.pt

Supplementary Material

**Supplementary Table S9**. Primers used to amplify and sequence the *norA* gene of clinical isolates.

| **Gene** | **Primers** | **Nucleotide sequence (5’-3’)** | **Amplicon size (bp)** | **Reference** |
| --- | --- | --- | --- | --- |
| ***S. epidermidis*** |  |  |  |  |
| *norA (a)* | norA_SE_ext_Fw | CAGATGTGAAAGCGAAGAAA | 817 | Costa et al., 2018 |
|  | norA_SE_int_Rv | CAACAACACCTAATGTTCCAG |  | Costa et al., 2018 |
| *norA (b)* | norA_SE_int_Fw | CCAGGAGCTGATAAAGCTAA | 1127 | Costa et al., 2018 |
|  | norA_SE_Rv2^(1)^ | ATCTCYTTACCCACATTTCTC |  | This work |
| ***S. haemolyticus*** |  |  |  |  |
| *norA (a)* | norA_Sha_A_Fw | GTCTAGCACTAAAAGAAACG | 1123 | This work |
|  | norA_Sha_A_Rv | GCTGAATAGAGTAATGACCAG |  | This work |
| *norA (b)* | norA_Sha_B_Fw | CGCCAATTATTTTGACATTAG | 733 | This work |
|  | norA_Sha_B_Rv | TAACTGTAATCTTTGTAATACTTCT |  | This work |

**Supplementary Table S9**. Continued.

| ***S. hominis*** |  |  |  |  |
| --- | --- | --- | --- | --- |
| *norA (a)* | norA_Sho_A_Fw | TCTCAGAATATGAATTAGATGAAT | 889 | This work |
|  | norA_Sho_A_Rv | CAAGTAATAAATGTAAGTTCAGTG |  | This work |
| *norA (b)* | norA_Sho_B_Fw | TACTTCAGCGAAAGCAAACTAC | 644 | This work |
|  | norA_Sho_B_Rv | TTCATCTCCTTACCCACACTTC |  | This work |

^(1)^ In case of no amplification, an alternative reverse primer was used (AAGAAGTGGGACACAGAATT), resulting in an amplicon with 912 bp

**Supplementary Table S10**. Conditions for *norA* gene amplification in clinical isolates of *S. epidermidis*, *S. haemolyticus* and *S. hominis.*

| **Species** | **Gene** | **Amplification conditions** | **Gene** | **Amplification conditions** |
| --- | --- | --- | --- | --- |
| ***S. epidermidis*** | *norA (a)* | 95°C – 3 min  94°C – 1 min  57°C – 1 min 35 cycles  72°C – 1 min  72°C – 5 min | *norA (b)*^(1)^ | 95°C – 3 min  94°C – 1 min  59°C – 45 sec 35 cycles  72°C – 90 sec  72°C – 5 min |
| ***S. haemolyticus*** | *norA (a)* | 95°C – 3 min  94°C – 45 sec  51°C – 45 sec 30 cycles  72°C – 1 min  72°C – 5 min | *norA (b)* | 95°C – 3 min  94°C – 30 sec  50°C – 40 sec 30 cycles  72°C – 40 sec  72°C – 5 min |
| ***S. hominis*** | *norA (a)* | 95°C – 3 min  94°C – 30 sec  50°C – 45 sec 35 cycles  72°C – 45 sec  72°C – 5 min | *norA (b)* | 95°C – 3 min  94°C – 30 sec  56°C – 45 sec 30 cycles  72°C – 45 sec  72°C – 5 min |

^(1)^ For the alternative reverse primer (see Supplementary Table S9), the annealing temperature was 57°C.

**Supplementary Figure S1**. Complete multiple alignment of *S. aureus norBI* and *norBII* sequences. The three sequences are representative of *norBI* with 463 (*S. aureus* NCTC 8325) and 466 (*S. aureus* Be62) amino acids, and of *norBII* (*S. aureus* CA-347). The corresponding polypeptide sequence of *norBI* NCTC 8325 is shown above the alignment. The shaded regions correspond to the approximate positions of the transmembrane segments, predicted with DeepTMHMM 1.0 (Hallgren et al., 2022) and Phobius (Käll et al., 2007), using the NorBI sequence of *S. aureus* NCTC 8325. The amino acid regions inside the boxes are relative to the conserved motifs described for MFS transporters with 14 TMS. The red and blue residues shown above the main polypeptide sequence represent the variations present in the polypeptide sequences of NorBI Be62 and NorBII CA-347, respectively, relatively to the NorBI of NCTC 8325.

**(A)**

**(B)**

**Supplementary Figure S2.** Comparison of transmembrane regions and domains of (A) SACOL0163, (B) SACOL1982 and (C) SACOL2566 *S. aureus* COL MDR transporters with the putative homologous transporters of *S. epidermidis* ATCC12228, *S. haemolyticus* JCSC1435 and *S. lugdunensis* HKU09-01 with InterPro.

**(C)**

**Supplementary Figure S2.** Continued.
